# Supplementary figures and images for: Multiple-Cycle Polymeric Extracellular Vesicle Precipitation and Its Evaluation by Targeted Mass Spectrometry
Source: Int J Mol Sci. 2021 Apr 21;22(9):4311. doi: 10.3390/ijms22094311 (PMC8122279; doi:10.3390/ijms22094311)

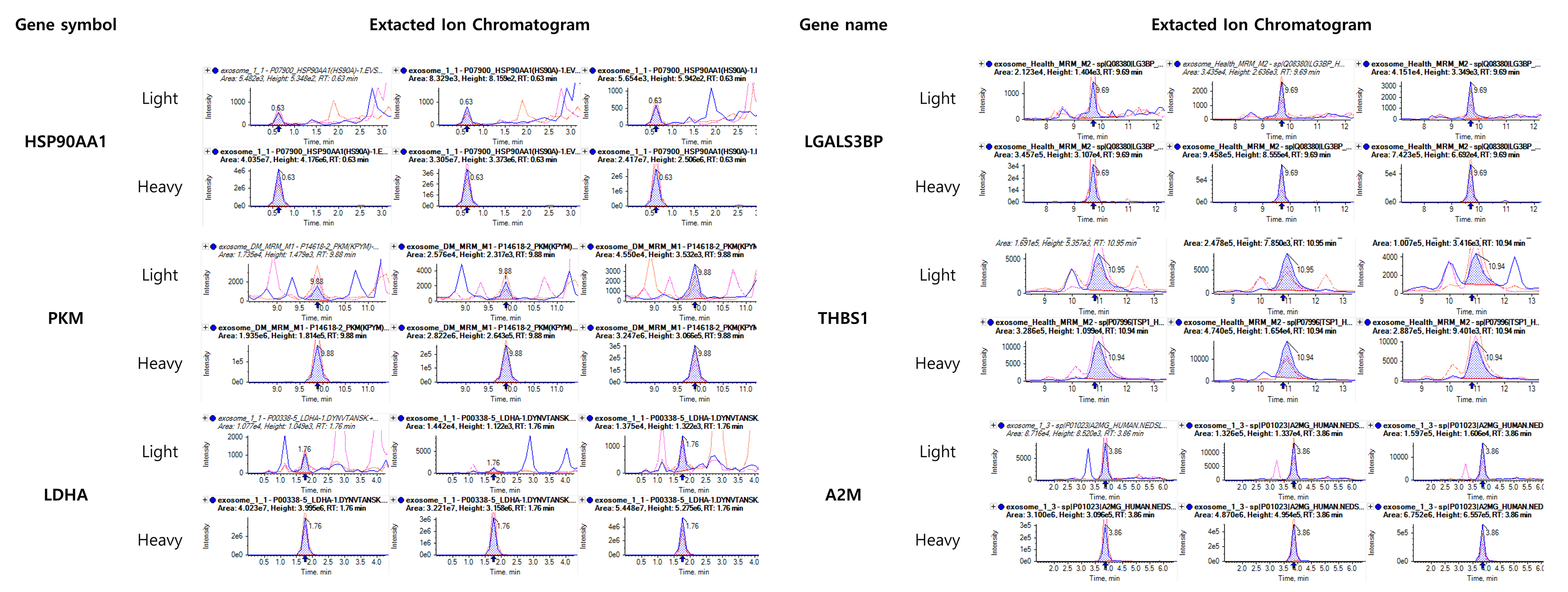

Supplement: Supplementary file 1 [file ijms-22-04311-s001.zip › Supporting information Fig._1.tif]

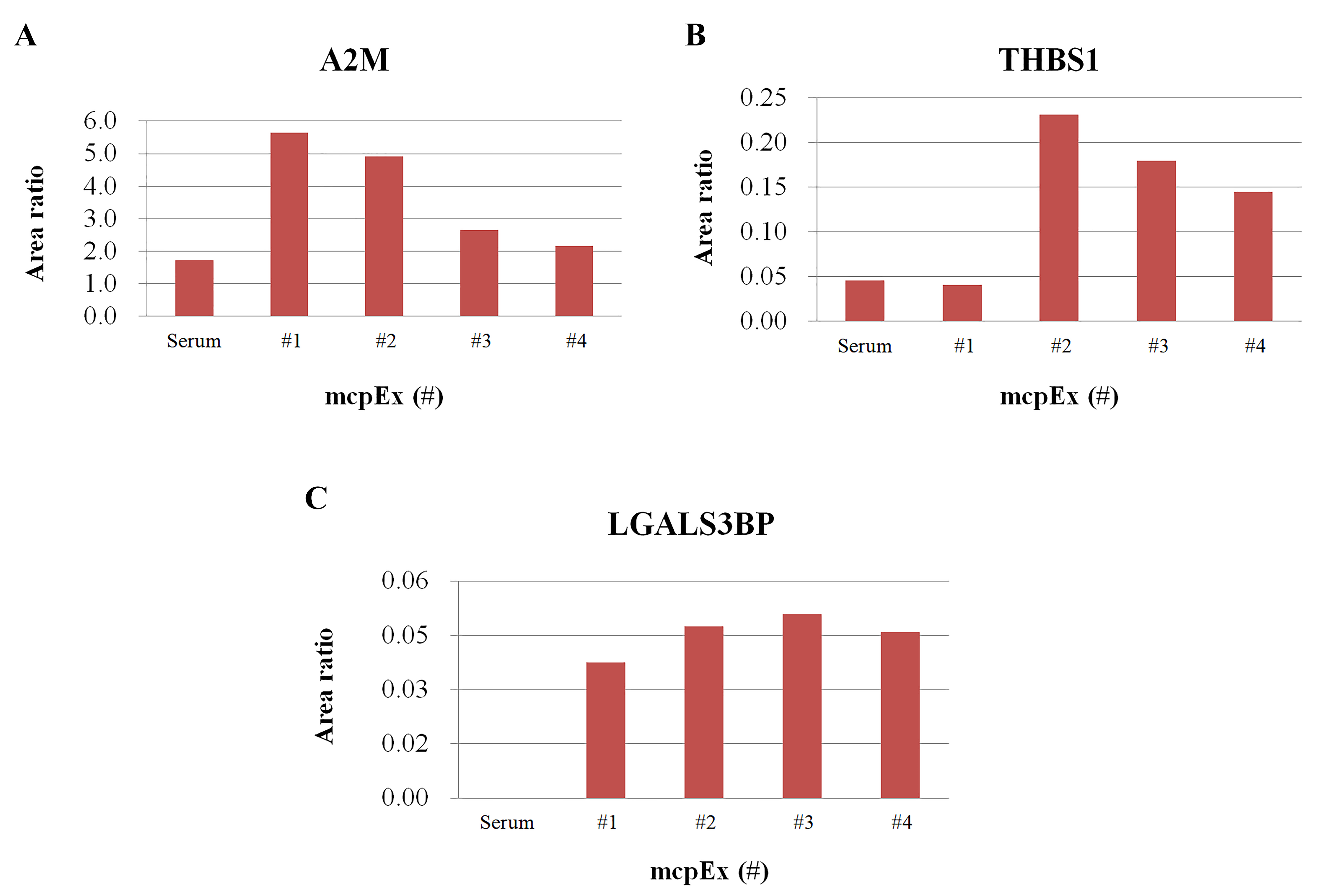

Supplement: Supplementary file 1 [file ijms-22-04311-s001.zip › Supporting information Fig._2.tif]
